# Hypercoagulable state and gut microbiota dysbiosis as predictors of poor functional outcomes in acute ischemic stroke patients

**DOI:** 10.1128/msystems.01492-24

**Published:** 2025-04-09

**Authors:** Jie Li, Shengnan Chen, Siqi Yang, Wen Zhang, Xiaoqi Huang, Lang Zhou, Yanchao Liu, Mengxi Li, Yonghui Guo, Jia Yin, Kaiyu Xu

**Affiliations:** 1 Microbiome Medicine Center, Department of Laboratory Medicine, Zhujiang Hospital, Southern Medical University, Guangzhou, Guangdong, China; 2 Department of Neurosurgery Center, Zhujiang Hospital, Southern Medical University, Guangzhou, Guangdong, China; 3 Department of Neurology, Nanfang Hospital, Southern Medical University, Guangzhou, Guangdong, China; Southern Medical University, Guangzhou, Guandong, China

**Keywords:** acute ischemic stroke, hypercoagulable state, gut microbiota, coagulation indices, functional outcome

## Abstract

Stroke is the second leading cause of death worldwide. Acute ischemic stroke (AIS) patients often exhibit hypercoagulable state and gut microbiota dysbiosis. However, the association between coagulation abnormalities and gut microbiota dysbiosis in AIS patients and their predictive value for poor functional outcomes in AIS has not been investigated. Our study enrolled 95 AIS patients and 81 healthy controls, using 16S rRNA sequencing to analyze gut microbiota composition. Baseline fibrinogen level was found to be an independent risk factor for poor functional outcomes at 90-day follow-up (odds ratio = 2.16, 95% confidence interval: 1.02–4.59, *P* = 0.044). AIS patients showed significant gut microbiota dysbiosis, with significantly increased *Parabacteroides* and *Alistipes*, and decreased *Prevotella* and *Roseburia*, associated with coagulation indices. Furthermore, compared with AIS patients with normal coagulation function, those in a hypercoagulable state exhibited a significant increase in *Alistipe*s and a decrease in *Prevotella*. We identified gut microbial biomarkers consisting of 15 bacteria that predicted poor functional outcome in AIS patients at 90-day follow-up. Coagulation indices improved the predictive performance of these biomarkers. In training and validation cohorts, area under the curve (AUC) values were 0.930 and 0.890 for microbial biomarkers alone, 0.691 and 0.751 for coagulation indices alone, and 0.943 and 0.944 for coagulation indices combined with gut microbial biomarkers. Our study showed that AIS patients with hypercoagulable state had gut microbiota dysbiosis, with *Alistipes* and *Prevotella* significantly associated with coagulation indices. A classification model based on coagulation indices and gut microbial biomarkers accurately predicted poor functional outcome in AIS patients at 90-day follow-up.

**IMPORTANCE:**

Acute ischemic stroke (AIS) patients often exhibit hypercoagulable state and gut microbiota dysbiosis. However, the relationship between hypercoagulable state and gut microbiota dysbiosis in AIS patients and their predictive value for poor functional outcomes has not been fully explored. Our study of 95 AIS patients showed that baseline fibrinogen level was an independent risk factor for poor functional outcome at 90-day follow-up in AIS patients. Hypercoagulable state in AIS patients correlates with gut microbiota dysbiosis. AIS patients with hypercoagulable state had increased *Alistipes* abundance and decreased *Prevotella* abundance. A classification model based on coagulation indices and gut microbial biomarkers accurately predicted poor functional outcome in AIS patients at 90-day follow-up.

## INTRODUCTION

In 2019, there were approximately 101 million stroke patients worldwide, with 12.2 million new stroke cases and 6.55 million stroke-related deaths (1). The incidence of stroke is increasing annually due to the increasing prevalence of risk factors such as hypertension, hyperlipidemia, obesity, and hyperglycemia (2, 3). This makes stroke the second leading cause of death and the third leading cause of disability worldwide (1, 4). Ischemic stroke accounts for approximately 62.4% of stroke cases and 50.2% of stroke-related deaths and is characterized by high morbidity, disability, mortality, and recurrence (1, 5, 6). Therefore, early identification of risk factors associated with the prognosis of acute ischemic stroke (AIS) patients is critical.

Coagulation indices are clinically relevant in AIS patients. Coagulation function is activated in AIS patients, with significantly elevated levels of fibrinogen and D-dimer (7, 8). On the one hand, coagulation indices may influence the clinical therapeutic management of AIS patients. In stroke patients anticoagulated with warfarin, recurrence of AIS can be effectively prevented when the international normalized ratio reaches 2.0–3.0 (9). On the other hand, coagulation indices are associated with complications in AIS patients, and AIS patients with abnormal coagulation indices may develop atrial fibrillation, malignancy, and venous thrombosis (10). At present, there are only observational studies on the hypercoagulable state of AIS patients, and the mechanism of coagulation abnormality after stroke onset needs further investigation.

Acute ischemic stroke causes gut microbiota dysbiosis, with an increase in opportunistic pathogens and a decrease in beneficial bacteria. Gut microbial dysbiosis may exacerbate brain injury after stroke and is associated with a worse prognosis after stroke. A specific gut microbiota index, calculated based on the difference in gut microbiota between AIS patients and healthy controls, is an independent predictor of severe stroke (11). Stroke-associated pneumonia (SAP)-related gut dysbiosis almost doubles the risk of SAP (12). In addition, the gut metabolite trimethylamine oxide (TMAO) increases the risk of thrombosis in AIS by mediating platelet hyperreactivity (13). TMAO also induces tissue factor expression in vascular endothelial cells, which promotes thrombus formation in atherosclerotic thrombi (14). However, whether the gut microbiota influences coagulation abnormalities in patients with AIS remains unexplored.

Therefore, in this study, we conducted a clinical study including 95 AIS patients and 81 healthy controls and analyzed the coagulation function of AIS patients and its association with gut microbiota. Our study also combined coagulation indices with gut microbial biomarkers to establish a predictive model that can accurately predict poor functional outcomes in AIS patients, which are beneficial for the early identification of AIS patients with worse prognosis, thus optimizing stroke management and improving stroke prognosis.

## MATERIALS AND METHODS

### Study design and participants

This observational clinical study was approved by the Ethics Committee of Zhujiang Hospital of Southern Medical University (2023-KY-112-02). Written informed consent was obtained from all participants. In this study, 95 patients with acute ischemic stroke were recruited from Zhujiang Hospital of Southern Medical University between March 2023 and March 2024. Inclusion criteria were as follows: (i) age over 18 years; (ii) diagnosed with acute ischemic stroke (15); (iii) admitted within 7 days after stroke onset. Exclusion criteria were as follows: (i) prior thrombolytic therapy before admission; (ii) inability to provide a stool sample within 3 days of admission; (iii) use of antibiotics, prebiotics, or probiotics within 1 month before admission; (iv) history of severe liver or kidney disease and malignant tumor; and (v) inability to complete the 90-day follow-up after discharge. The control group, including 81 healthy controls, was recruited from the Health Examination Center of Zhujiang Hospital of Southern Medical University. Inclusion criteria were as follows: (i) age and gender matched with the stroke patients; (ii) willing to provide fresh fecal samples. Exclusion criteria for healthy controls were the same as described above.

### Clinical data and sample collection

Baseline clinical data, including age, sex, stroke risk factors (hypertension, diabetes, coronary heart disease, and smoking), and laboratory results were collected from AIS patients. Neurological function scores, including National Institutes of Health Stroke Scale (NIHSS) score and modified Rankin Scale (mRS) score, were assessed by specialized neurologists at admission, discharge, and 90-day follow-up (16). Demographic information and laboratory data were also collected from healthy controls. Initial fecal samples were collected from the AIS group (*N* = 95) within 3 days of admission and from healthy controls (*N* = 81) and stored at −80°C. Plasma samples were collected from the healthy controls with normal coagulable function (CON-NC, *N* = 39), the AIS patients with normal coagulable function (AIS-NC, *N* = 24), and the AIS patients with hypercoagulable state (AIS-HC, *N* = 12). Whole blood samples were collected and centrifuged at 1,500 g for 10 minutes, and the top layer of plasma was collected and stored at −80°C.

### 16S rRNA sequencing and microbiome data analysis

Bacterial genomic DNA was extracted from fecal samples using the QIAGEN Fast Stool DNA Mini Kit according to the manufacturer’s instructions. The V3–V4 variable region of the 16S rRNA gene was amplified by PCR using primers V3–V4F (5'ACTCCTACGGGGAGGCAGCA3') and V3–V4R (5'GGACTACHVGGGGTWTCTAAT3') with barcode. All PCR amplicons were combined and sequenced on the Illumina NovaSeq 6000 platform. The quality of the raw sequencing data was checked using Fastp (version 0.14.1) (17). The “cutadapt” software was used to remove adapter sequences in the V3–V4 region of the 16S rRNA amplicon reads. The R package DADA2 (version 1.6.0) was used to denoise each paired-end fastq file to obtain amplicon sequence variants (18). Representative sequences were aligned using the PyNAST algorithm, and a phylogenetic tree was constructed using FastTree (19, 20). Taxonomic analysis was performed using the Ribosome Database Project Classifier based on the Greengenes database (version 13.8) (21, 22). Alpha diversity was calculated including Chao1 index, ACE index, Shannon index, and Simpson index. Beta diversity was calculated based on Bray-Curtis, unweighted Unifrac, and weighted Unifrac distance and presented in principal coordinates analysis (PCoA) plots. Differential bacterial genera were identified using linear discriminant analysis effect size (LEfSe; Wilcoxon < 0.05, Kruskal-Wallis < 0.05, and linear discriminant analysis (LDA) score > 3). Functional prediction analysis of 16S rRNA sequencing data based on the MetaCyc database was performed using PICRUSt2 software (23).

### Coagulation factor testing

Coagulation factor testing was performed in the CON-NC, AIS-NC, and AIS-HC groups. Von Willebrand factor (VWF) and coagulation factors (factors VII, VIII, IX, XI, and XII) were measured using the ACL TOP700 fully automated coagulation analyzer (Werfen, America). VWF activity level (VWF:C) was determined by immunoturbidimetric assay. Coagulation factor activity level (F:C) was determined by a one-step coagulation assay (24).

### Statistical analysis

Statistical analysis was performed with IBM SPSS 26.0 software and R (version 4.2.1). Continuous variables were presented as medians (interquartile range, IQR), and categorical variables were presented as frequencies (percentages). Missing values were interpolated using the median within 5% for all variables. Clinical data were compared using the Wilcoxon rank-sum test or the Kruskal-Wallis test for continuous variables and the *χ*^2^ test or the Fisher exact test for categorical variables. Odds ratio (OR) and 95% confidence interval (CI) were calculated by univariate and multivariate logistic regression analysis to explore risk factors associated with poor functional outcomes in AIS patients at 90-day follow-up. Spearman correlation analysis was used to determine the correlation of coagulation indices with mRS scores and gut microbiota in AIS patients, as well as the correlation of differential functional pathways with gut microbiota between the AIS-NC and AIS-HC groups. The importance of different microbial genera between AIS patients and healthy controls was ranked by the mean decrease Gini index in the random forest algorithm using the RandomForest software package for R (version 4.2.1). The best combination of gut microbial biomarkers was identified using a random forest algorithm through five repeats of 10-fold cross-validation. To assess the model’s stability and reproducibility, the average receiver operaing characteristic (ROC) curve with its 95% confidence interval (CI) was calculated based on the aforementioned cross-validation results. Predictive models were constructed using multifactorial logistic regression, and ROC curves were plotted. The predictive ability of the model was calculated from the AUC of the subject work characteristic for both the training and validation sets. *P* < 0.05 was considered statistically significant.

## RESULTS

### Baseline characteristics of the study participants

**Fig 1:**
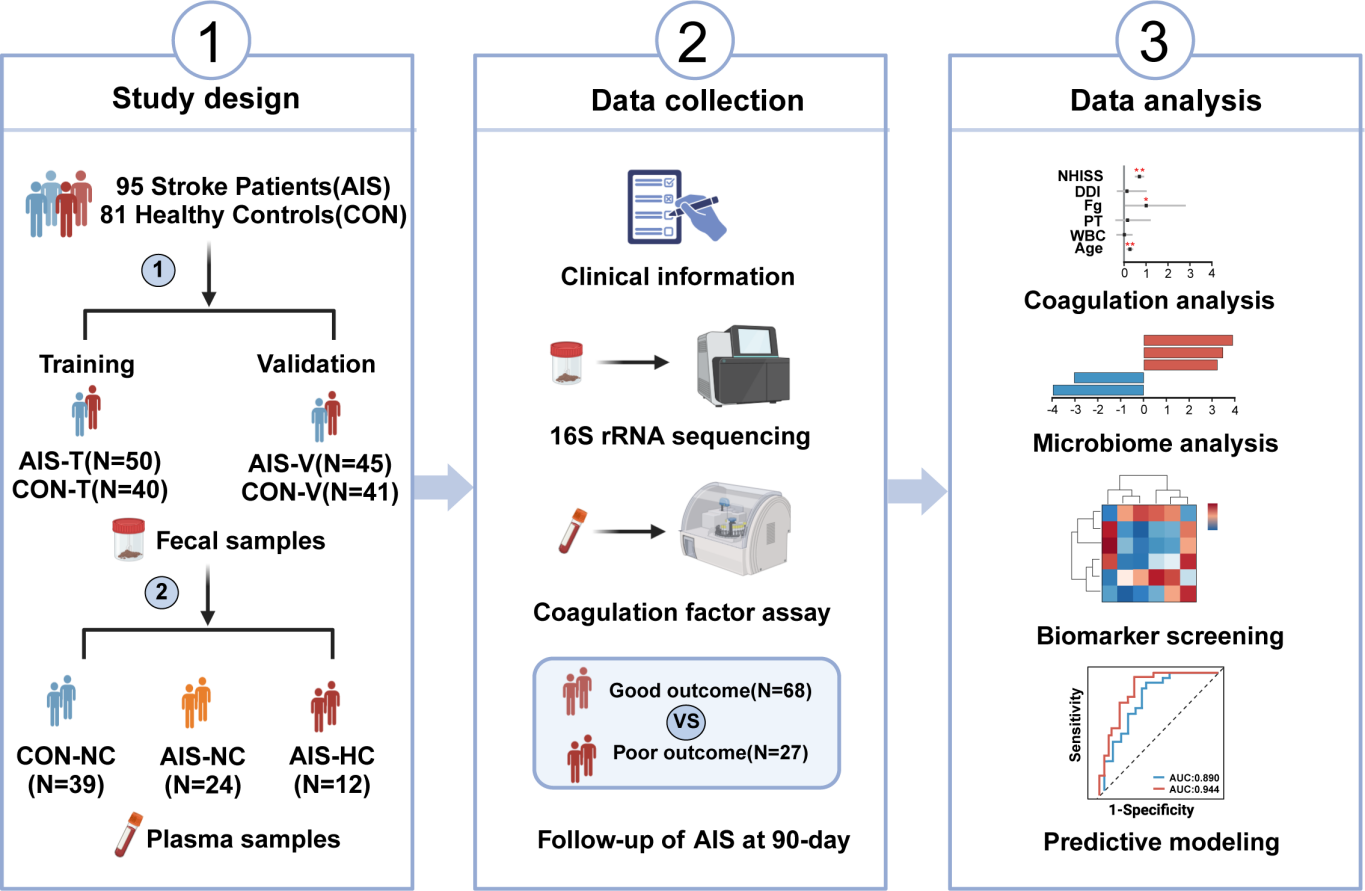
Flow chart of this study. Image drawn from Biorender.com. CON, healthy controls; AIS-T, AIS patients in training cohort; CON-T, healthy controls in training cohort; AIS-V, AIS patients in validation cohort; CON-V, healthy controls in validation cohort; CON-NC group, healthy controls with normal coagulable function; AIS-NC group, AIS patients with normal coagulable function; AIS-HC group, AIS patients with hypercoagulable state.

A total of 95 patients with acute ischemic stroke and 81 healthy controls were enrolled in this study from March 2023 to March 2024 at Zhujiang Hospital, Southern Medical University (Fig. 1). Age and gender were comparable between AIS patients and healthy controls. AIS patients had a higher prevalence of hypertension and diabetes mellitus compared with healthy controls, while smoking history and coronary heart disease history were comparable. In addition, white blood cells (WBC), glucose, fibrinogen, and D-dimer levels were significantly higher in AIS patients compared with healthy controls (Table 1). All patients in the AIS group completed the 90-day follow-up using the mRS scores. The follow-up results showed that 27 patients with AIS had poor functional outcomes (mRS > 2), and 68 patients with AIS had good functional outcomes (mRS ≤ 2; Table S1).

**TABLE 1:** Characteristics of the study participants^*a*^

Characteristics	Healthy controls	AIS patients	*P* value
Number	81	95	
Age, years	66.00 (12.00)	67.00 (16.00)	0.173
Male, *n* (%)	44 (54.32)	57 (60.00)	0.448
Hypertension, *n* (%)	27 (33.33)	67 (70.53)	<0.001
Diabetes, *n* (%)	5 (6.17)	25 (26.32)	<0.001
Coronary heart disease, *n* (%)	1 (1.23)	7 (7.37)	0.113
Smoking, *n* (%)	5 (6.17)	11 (11.58)	0.214
WBC, 10^9^/L	6.38 (2.76)	7.72 (3.66)	0.002
RBC, 10^12^/L	4.49 (0.61)	4.61 (0.80)	0.236
HGB, g/L	134.00 (19.50)	136.00 (23.00)	0.715
PLT, 10^9^/L	229.00 (77.00)	228.00 (75.00)	0.312
ALT, IU/L	16.00 (8.00)	15.00 (10.00)	0.214
AST, IU/L	18.00 (7.00)	18.00 (7.00)	0.981
Cr, μmol/L	72.00 (24.50)	76.00 (26.00)	0.343
Glu, mmol/L	5.15 (0.88)	6.50 (3.31)	<0.001
PT, s	11.30 (0.90)	11.50 (1.20)	0.468
APTT, s	25.90 (2.30)	25.40 (3.00)	0.407
TT, s	16.10 (0.85)	16.00 (1.20)	0.433
DDI, mg/L	0.32 (0.25)	0.38 (0.60)	0.040
Fg, g/L	2.83 (0.87)	2.97 (1.25)	0.013

^*a*^ Continuous variables are expressed as medians (IQR), and categorical variables are expressed as frequencies (percentages). RBC, red blood cells; HGB, hemoglobin; PLT, platelets; ALT, alanine aminotransferase; AST, aspartate transaminase; Cr, creatinine; Glu, glucose; PT, prothrombin time; APTT, activated partial thromboplastin time; TT, thrombin time; DDI, D-dimer; Fg, fibrinogen.

### Hypercoagulable state is a risk factor for poor functional outcomes in AIS patients

**Fig 2:**
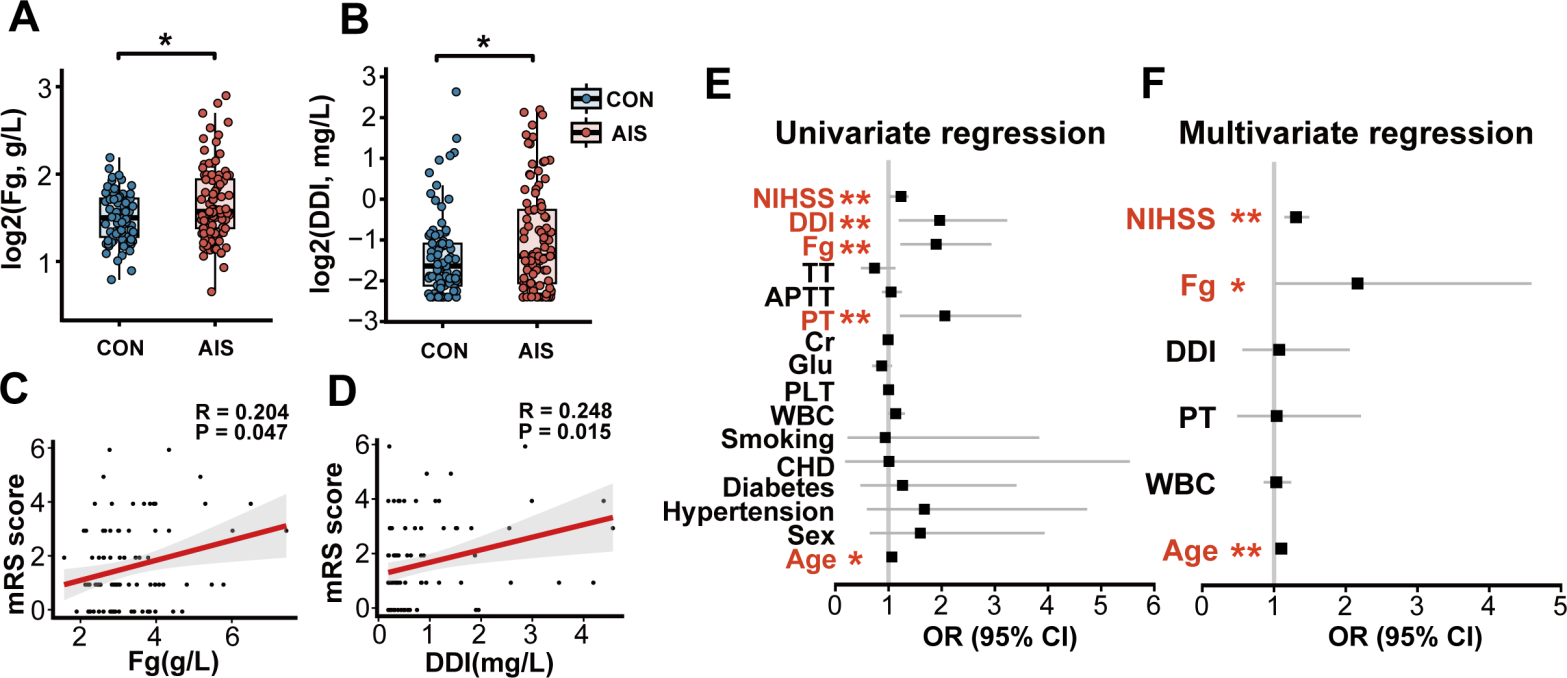
AIS hypercoagulable state is associated with poor functional outcome. (**A and B**) Fibrinogen and D-dimer levels significantly discriminated between AIS patients and healthy controls. (**C and D**) Fibrinogen and D-dimer levels were significantly positively correlated with mRS score in patients with AIS. (**E**) Univariate logistic regression analysis of risk factors for poor functional outcome in patients with AIS. (**F**) Multivariate logistic regression of risk factors for poor functional outcome in patients with AIS after adjustment for confounders. CHD, coronary heart disease; PLT, platelets; Glu, glucose; Cr, creatinine; APTT, activated partial thromboplastin time; DDI, D-dimer; Fg, fibrinogen. CON, healthy controls. *P < 0.05 and **P < 0.01.

The AIS patients showed a hypercoagulable state with significantly elevated levels of fibrinogen and D-dimer compared to the control group (Fig. 2A and B). Spearman’s correlation analysis showed a significant positive correlation between the levels of fibrinogen and the mRS scores of the AIS patients at 90-day follow-up (Fig. 2C). Similarly, the levels of D-dimer and the mRS scores of the AIS patients at 90-day follow-up also showed a significant positive correlation (Fig. 2D). To investigate the association between fibrinogen and D-dimer and poor functional outcomes in AIS patients, we categorized AIS into a good functional outcome group (mRS ≤ 2, *N* = 68) and a poor functional outcome group (mRS > 2, *N* = 27) based on mRS scores at 90-day follow-up. Baseline characteristics of AIS patients with good and poor outcomes are shown in Table S1. Compared to the good outcome group, the poor outcome group had significantly longer prothrombin time (PT), significantly shorter thrombin time (TT), and significantly higher fibrinogen and D-dimer levels (Table S1). The results of univariate logistic regression analysis showed that fibrinogen and D-dimer were risk factors for poor functional outcomes in AIS patients (Fig. 2E; Table S2). In the multivariate logistic regression analysis, the significance of D-dimer disappeared and fibrinogen retained its significance (OR = 2.16, 95% CI: 1.02–4.59, *P* = 0.044) after correcting for the risk factors with *P* < 0.1 in the univariate regression analysis (age, WBC, PT, D-dimer, and NIHSS; Fig. 2F; Table S2). Our results suggest that fibrinogen levels in the acute phase of stroke patients are an independent risk factor for poor functional outcome in stroke patients at 90-day follow-up.

### Gut microbiota dysbiosis associated with hypercoagulable state in AIS patients

**Fig 3:**
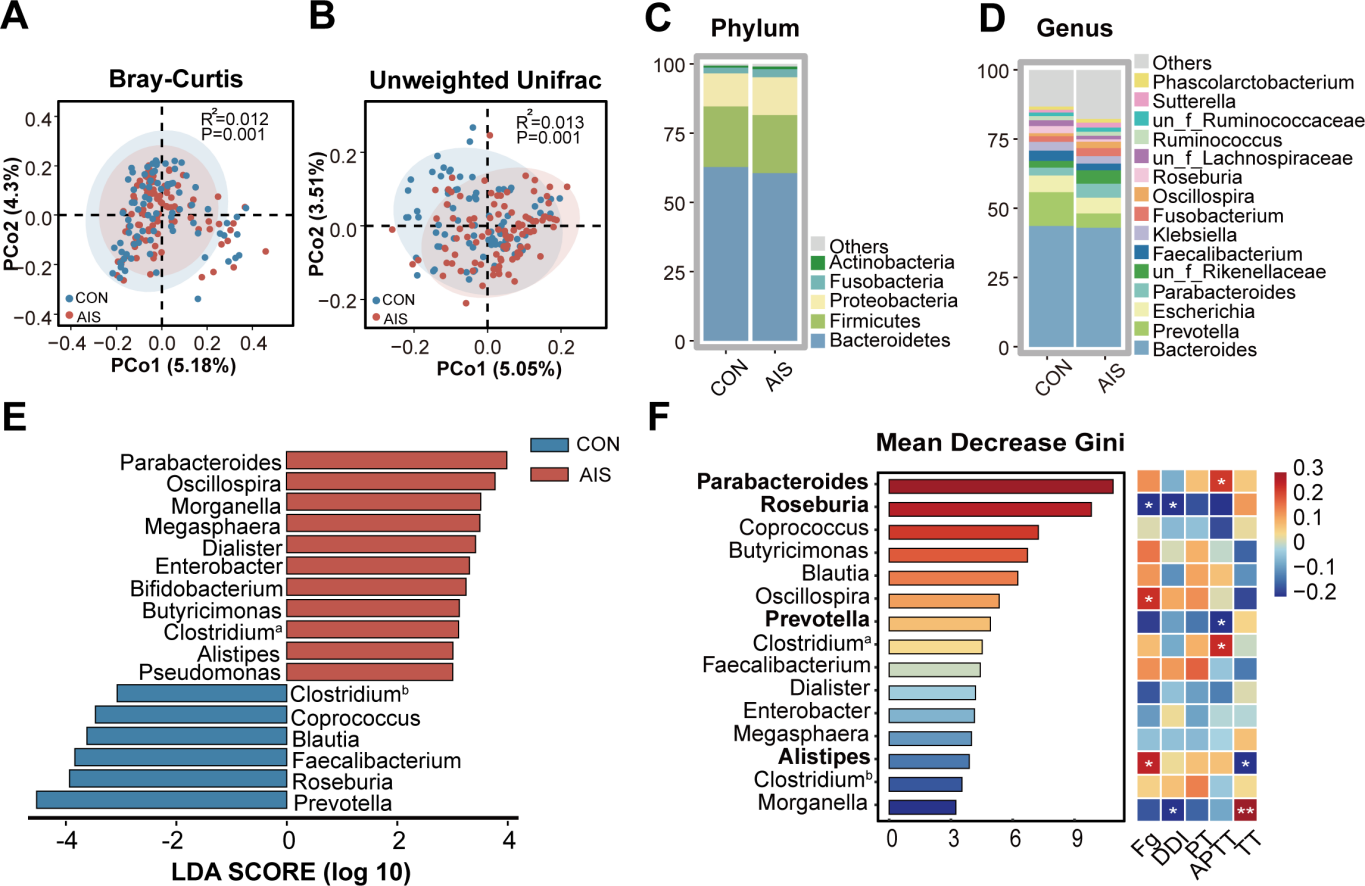
Significantly altered gut microbiota composition in AIS patients correlates with coagulation indices. (**A and B**) PCoA plots based on Bray-Curtis and unweighted Unifrac showing significant differences in gut microbiota composition between AIS patients and healthy controls. (**C and D**) Relative abundance of gut microbiota at the phylum and genus level in AIS patients and healthy controls. (**E**) LEfSe analysis showing differential genera between AIS patients and healthy controls (Wilcoxon < 0.05, Kruskal-Wallis < 0.05, and LDA > 3). (**F**) Random forest algorithm screening the top 15 differential genera between AIS patients and healthy controls, and Spearman correlation analysis with coagulation indices in AIS patients. Clostridium^a^, Clostridium_f_Lachnospiraceae; Clostridium^b^, Clostridium_f_Clostridiaceae. Fg, fibrinogen; DDI, D-dimer; APTT, activated partial thromboplastin time. CON, healthy controls. *P < 0.05 and **P < 0.01.

Using 16SrRNA sequencing, we analyzed the differences in gut microbiota composition between AIS patients and healthy controls. Alpha-diversity analysis showed that Chao1 and ACE index were significantly higher in AIS patients compared to healthy controls, while there was no significant difference in Shannon and Simpson indices (Fig. S1). PCoA plot based on Bray-Curtis (*R*^2^ = 0.012, *P* = 0.001) and unweighted Unifrac (*R*^2^ = 0.013, *P* = 0.001) both showed significant differences in gut microbiota composition between AIS patients and healthy controls (Fig. 3A–B). At the phylum level, the gut microbiota was dominated by Bacteroidetes, Firmicutes, and Proteobacteria (Fig. 3C; Fig. S2A), while at the genus level, the most abundant bacteria were *Bacteroides*, *Prevotella*, *Escherichia*, and *Parabacteroides* in both AIS and control groups (Fig. 3D; Fig. S2B). LEfSe analysis showed that *Parabacteroides*, *Oscillospira*, *Morganella*, *Megasphaera*, and *Alistipes* were significantly enriched in AIS patients, whereas *Prevotella*, *Roseburia*, *Faecalibacterium*, and *Blautia* were significantly enriched in healthy controls (Fig. 3E).

To identify the most significant intestinal bacteria distinguishing AIS patients from healthy controls, 15 bacteria screened by LEfSe analysis were ranked by importance using the Random Forest algorithm (Fig. 3F). Specifically, *Parabacteroides*, *Roseburia*, and *Coprococcus* were the three most critical categorical variables for differentiating AIS patients from healthy controls. Spearman’s correlation analysis showed that *Parabacteroides*, which was enriched in AIS patients, was significantly positively correlated with activated partial thromboplastin time (APTT; *R* = 0.231, *P* = 0.025), and *Alistipes* was significantly positively correlated with fibrinogen (*R* = 0.259, *P* = 0.011). Conversely, *Prevotella*, which was significantly reduced in AIS patients, showed a significant negative correlation with APTT (*R* = −0.239, *P* = 0.02). *Roseburia* showed a significant negative correlation with fibrinogen (*R* = −0.243, *P* = 0.018) and D-dimer (*R* = −0.212, *P* = 0.04) (Fig. 3F). These results suggest that significant gut microbiota dysbiosis in AIS patients is associated with their hypercoagulable state indices.

### AIS patients with hypercoagulable state showed significantly increased *Alistipes* and decreased *Prevotella*

**Fig 4:**
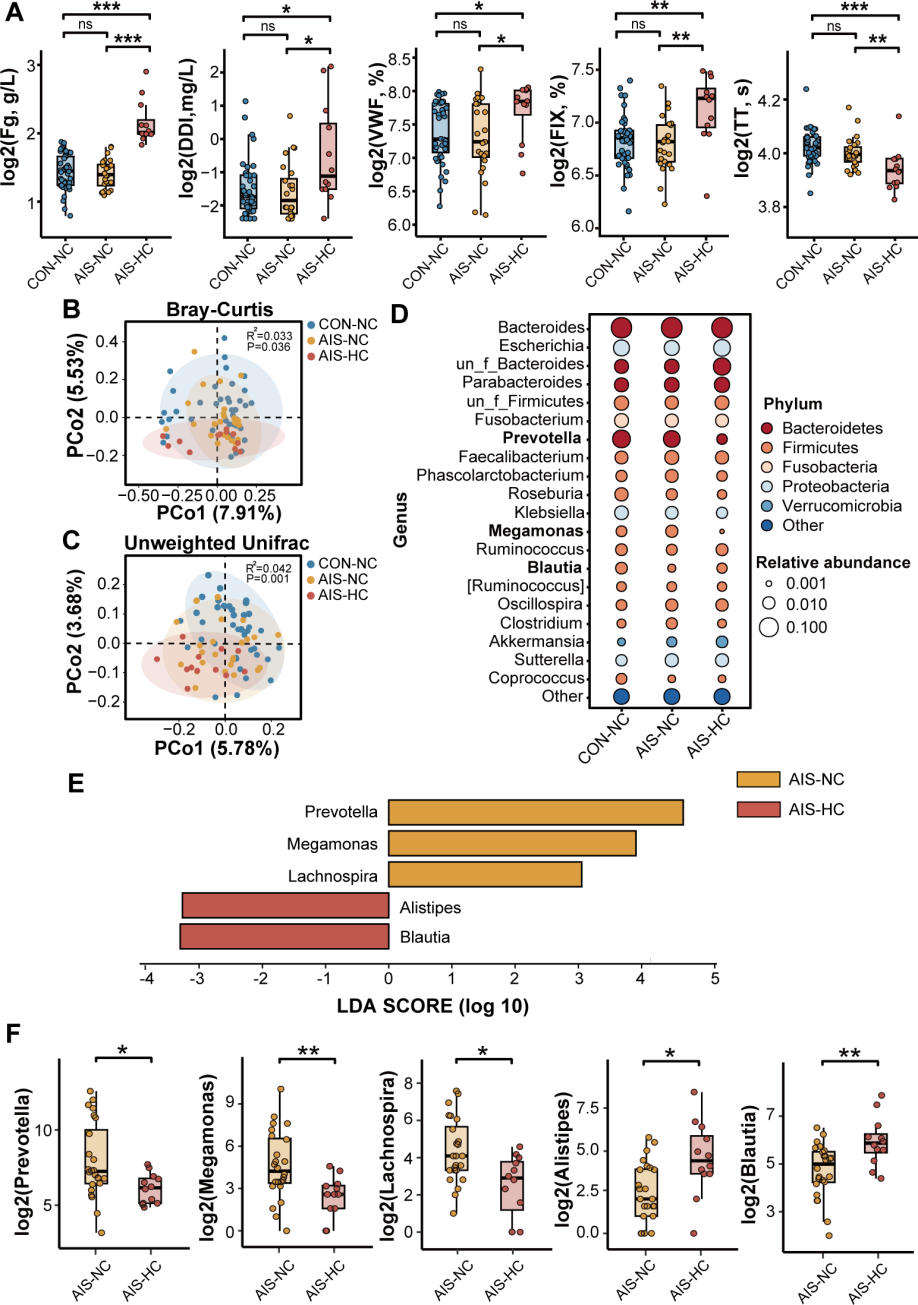
Gut microbiota composition among CON-NC, AIS-NC, and AIS-HC groups. (**A**) The plasma levels of Fg, DDI, VWF, FIX, and TT among the CON-NC, AIS-NC, and AIS-HC groups. (**B and C**) PCoA plots based on Bray-Curtis and unweighted Unifrac showing significant differences in gut microbiota composition among CON-NC, AIS-NC, and AIS-HC groups. (**D**) Bubble plots showing the relative abundance of major microbial genera in the CON-NC, AIS-NC, and AIS-HC groups. The color of the bubbles reflects the relationship between genera and phyla, and the size of the bubbles reflects the relative abundance of the genera. (**E**) LEfSe analysis screening for differential gut microbial bacteria between AIS-NC and AIS-HC groups. (**F**) The abundance of *Prevotella*, *Megamonas*, *Lachnospira*, *Alistipes*, and *Blautia* in the AIS-NC and AIS-HC groups. Fg, fibrinogen; DDI, D-dimer; FIX, coagulation factor IX. *P < 0.05, **P < 0.01, and ***P < 0.001.

To further investigate the relationship between gut microbiota dysbiosis and hypercoagulable state in AIS patients, we further analyzed the differentiation of gut microbiota composition between AIS patients with hypercoagulable state and AIS patients with normal coagulable function. A total of 36 AIS patients were divided into AIS-HC group (*N* = 12, Fg > 3.5 g/L) and AIS-NC group (*N* = 24, Fg ≤ 3.5 g/L) based on plasma fibrinogen levels (3.5 g/L). Thirty-nine age- and sex-matched healthy subjects (CON-NC, *N* = 39) were enrolled as controls. Plasma samples were collected from all subjects, and plasma coagulation factor levels were measured. No significant difference in coagulation factor levels was observed between the AIS-NC and CON-NC groups. In the AIS-HC group, plasma levels of fibrinogen, D-dimer, VWF, and coagulation factor IX were significantly higher, and TT was significantly shorter compared with both the CON-NC and AIS-NC groups (Fig. 4A; Table 2), indicating significantly activated coagulation function in AIS-HC group patients.

**TABLE 2:** Characteristics of study participants in the CON-NC, AIS-NC, and AIS-HC groups^*a*^

Characteristics	CON-NC	AIS-NC	AIS-HC	*P* value
Number	39	24	12	
Age, years	67.00 (10.00)	65.50 (13.75)	67.50 (12.50)	0.781
Male, *n* (%)	23 (58.97%)	17 (70.80%)	8 (66.60%)	0.622
WBC, 10^9^/L	6.08 (2.38)	6.87 (4.31)	9.47 (3.90)^*c*^	0.002
RBC, 10^12^/L	4.42 (0.69)	4.57 (0.97)	4.76 (1.15)	0.260
HGB, g/L	134.00 (17.00)	135.00 (19.50)	136.00 (41.75)	0.863
PLT, 10^9^/L	220.00 (89.00)	214.00 (82.50)	251.50 (139.75)	0.203
ALT, IU/L	16.00 (7.00)	15.00 (5.50)	15.00 (16.00)	0.950
AST, IU/L	18.00 (6.00)	17.50 (4.75)	16.00 (5.75)^*c*^	0.103
Cr, μmol/L	67.00 (24.00)	77.50 (21.91)	78.05 (46.13)	0.345
Glu, mmol/L	5.02 (0.79)	6.38 (3.30)^*b*^	6.82 (6.96)^*c*^	<0.001
PT, s	11.50 (0.70)	11.45 (0.97)	11.75 (1.15)	0.231
APTT, s	26.20 (2.10)	25.10 (2.70)	25.90 (5.42)	0.233
TT, s	16.20 (0.80)	15.95 (0.65)	15.30 (1.08)^*c*^*^,d^*	0.001
DDI, s	0.30 (0.24)	0.28 (0.26)	0.47 (1.33)^*c*^*^,d^*	0.037
Fg, g/L	2.74 (0.80)	2.63 (0.69)	4.03 (1.09)^*c*^*^,d^*	<0.001
VWF:Ag, %	157.00 (92.80)	151.90 (97.60)	230.45 (92.30)^*c*^*^,d^*	0.045
FVII, %	87.10 (23.20)	96.00 (27.70)	91.05 (48.93)	0.166
FVIII, %	126.80 (35.90)	138.00 (67.03)	180.80 (110.05)^*c*^	0.023
FIX, %	115.40 (19.60)	112.45 (27.95)	149.65 (47.83)^*c*^*^,d^*	0.003
FXI, %	101.20 (22.20)	100.75 (33.50)	120.40 (59.53)	0.269
FXII, %	50.70 (37.10)	56.25 (32.72)	53.40 (44.30)	0.939

^*a*^ Continuous variables are expressed as medians (IQR), and categorical variables are expressed as frequencies (percentages). RBC, red blood cells; HGB, hemoglobin; PLT, platelets; ALT, alanine aminotransferase; AST, aspartate transaminase; Cr, creatinine; Glu, glucose; DDI, D-dimer; Fg, fibrinogen; FVII, coagulation factor VII; FVIII, coagulation factor VIII; FIX, coagulation factor IX; FXI, coagulation factor XI; FXII, coagulation factor XII.

^*b*^ *P* < 0.05 when AIS-NC group compared with CON-NC group.

^*c*^ *P* < 0.05 when AIS-HC group compared with CON-NC group.

^*d*^ *P* < 0.05 when AIS-HC group compared with AIS-NC group.

We explored the gut microbiota composition among the AIS-HC, AIS-NC, and CON-NC groups. Alpha diversity in the AIS-HC group was comparable to the AIS-NC group. Simpson’s index was significantly higher in the AIS-HC group compared to the CON-NC group (Fig. S3A). PCoA plots based on Bray-Curtis and unweighted UniFrac showed significant differences in gut microbiota composition among the CON-NC, AIS-NC, and AIS-HC groups (Bray-Curtis: *R*^2^ = 0.033, *P* = 0.036; unweighted UniFrac: *R*^2^ = 0.042, *P* = 0.001; Fig. 4B and C). Bubble plots showed the dynamic changes of the 5 most abundant phyla and 20 most abundant genera among the AIS-HC, AIS-NC, and CON-NC groups (Fig. 4D). Bacteroidetes, Firmicutes, and Proteobacteria were the most abundant phyla (Fig. S3B), and *Bacteroides*, *Prevotella*, *Escherichia*, and *Parabacteroides* were the most abundant genera in the AIS-HC, AIS-NC, and CON-NC groups (Fig. S3C). More precisely, we explored the differences in gut microbiota between the AIS-NC and AIS-HC groups separately. β Diversity analysis revealed significant gut microbial differences between the AIS-NC and AIS-HC groups (Bray-Curtis: *R*^2^ = 0.037, *P* = 0.036; weighted UniFrac: *R*^2^ = 0.084, *P* = 0.004; Fig. S3D and E). LEfSe and Wilcoxon analyses showed significant enrichment of *Blautia* and *Alistipes* in the AIS-HC group compared to the AIS-NC group, while the abundance of *Prevotella*, *Megamonas*, and *Lachnospira* was significantly reduced (Fig. 4E and F).

**Fig 5:**
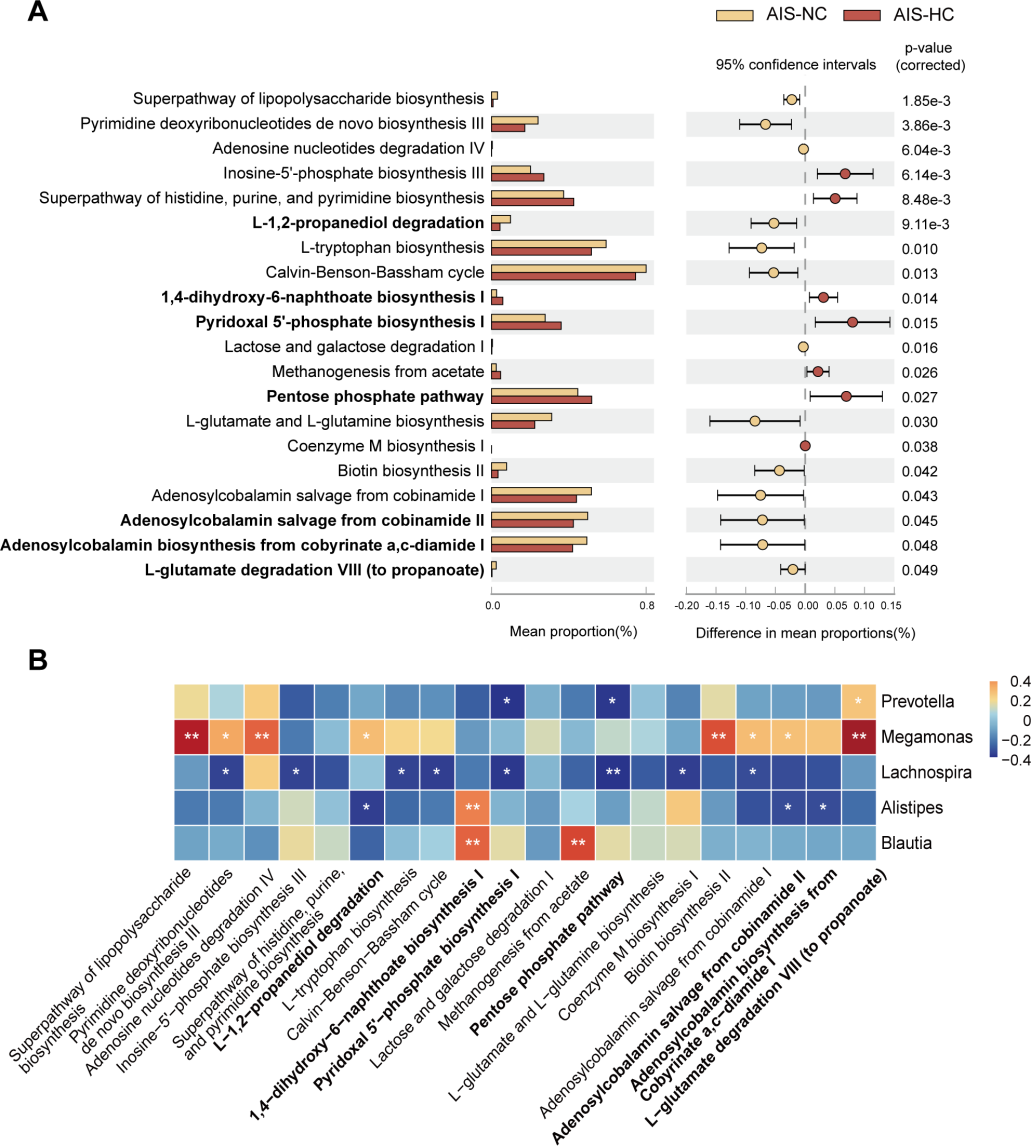
Significantly altered functional pathways between the AIS-NC and AIS-HC groups and their correlation with gut microbiota. (**A**) PICRUSt2 analysis of the gut microbial functional pathways between the AIS-NC and AIS-HC groups. (**B**) Spearman’s correlation analysis of significantly altered functional pathways with differential bacteria between AIS-NC and AIS-HC groups. *P < 0.05 and **P < 0.01.

Furthermore, we screened the differential functional pathways between the AIS-NC group and the AIS-HC group using PICRUSt2 analysis and explored their correlation with the above differential bacteria. Compared with the AIS-NC group, the 1,4-dihydroxy-6-naphthoate biosynthesis I, pyridoxal 5'-phosphate biosynthesis I, and pentose phosphate pathway were significantly up-regulated in the AIS-HC group (Fig. 5A). L-1,2-propanediol degradation, adenosylcobalamin salvage from cobinamide II, adenosylcobalamin biosynthesis from cobyrinate a,c-diamide I, and L-glutamate degradation VIII (to propanoate) pathways were significantly down-regulated in the AIS-HC group (Fig. 5A). Spearman correlation analysis showed that both *Prevotella* and *Megamonas*, which were significantly reduced in the AIS-HC group, were significantly and positively correlated with the L-glutamate degradation VIII (to propanoate) pathway (Fig. 5B). *Prevotella* and *Lachnospira*, which were significantly reduced in the AIS-HC group, were significantly and negatively correlated with pyridoxal 5'-phosphate biosynthesis I and pentose phosphate pathways (Fig. 5B). Both *Blautia* and *Alistipes*, significantly enriched in the AIS-HC group, were significantly positively associated with the 1,4-dihydroxy-6-naphthoate biosynthesis I pathway (Fig. 5B). *Alistipes* was also significantly negatively correlated with L-1,2-propanediol degradation, adenosylcobalamin salvage from cobinamide II, and adenosylcobalamin biosynthesis from cobyrinate a,c-diamide I pathways (Fig. 5B).

Therefore, AIS patients with hypercoagulable state showed increased *Alistipes* and decreased *Prevotella*, along with alterations in gut microbiota-related functional pathways.

### Combination of coagulation indices with gut microbial biomarkers shows the highest predictive accuracy for poor functional outcomes in AIS patients

**Fig 6:**
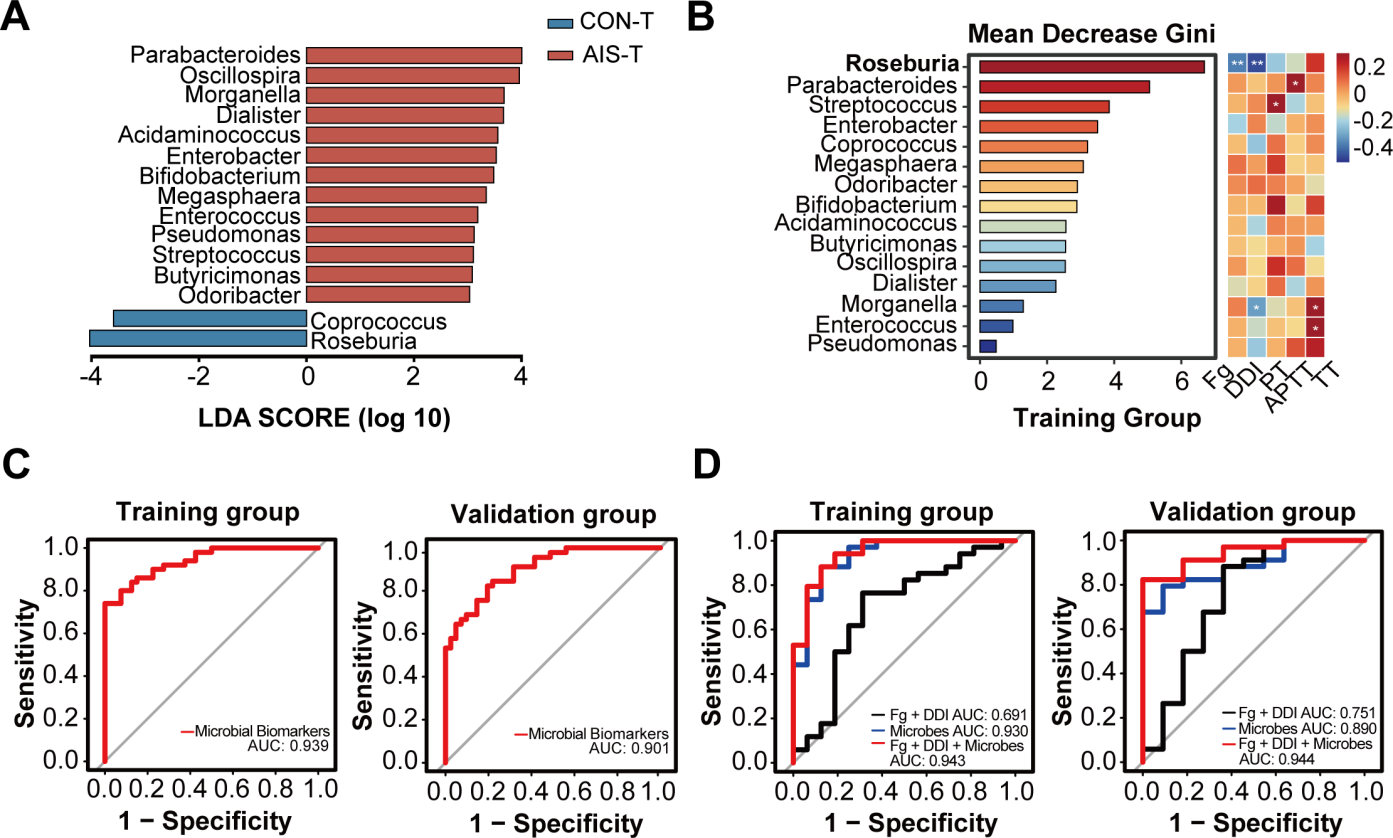
Coagulation markers could improve the efficacy of gut microbiota biomarkers in predicting clinical outcome in AIS patients. (**A**) LEfSe analysis showing differential microbial genera between AIS patients and healthy controls in the training cohort. (**B**) Random forest analysis of 15 differential gut microbial genera in AIS-T group and Spearman correlation analysis with clinical coagulation markers in AIS-T group. (**C**) ROC curves of logistic regression models of gut microbial biomarkers discriminating AIS patients from healthy controls in the training and validation cohorts. (**D**) ROC curves of logistic regression models of coagulation indices, gut microbial biomarkers, and microbial biomarkers with coagulation indices in predicting clinical outcome in AIS patients in the training cohort and validation cohort. Fg, fibrinogen; DDI, D-dimer. *P < 0.05 and **P < 0.01.

To build a predictive model for poor functional outcomes in AIS patients, we randomized the entire AIS cohort into a training set (AIS patients, *N* = 50 and healthy controls, *N* = 40) and a validation set (AIS patients, *N* = 45 and healthy controls, *N* = 41). Age and gender were comparable between AIS patients and healthy controls in both training and validation cohorts (Table S3). In the training cohort, alpha diversity indices, including Chao1 and ACE, were significantly higher in AIS patients compared to healthy controls (Fig. S4A). The composition of the gut microbiota was also significantly separated between the AIS patients in the training cohort (AIS-T) group and the healthy controls in the training cohort (CON-T) group based on the PCoA plot with Bray-Curtis (*R*^2^ = 0.017, *P* = 0.006) and unweighted Unifrac (*R*^2^ = 0.025, *P* = 0.001; Fig. S4B and C). At the phylum level, the most abundant bacteria were Bacteroidetes, Firmicutes, and Proteobacteria, while at the genus level, the most abundant bacteria were *Bacteroides*, *Prevotella*, *Escherichia*, and *Parabacteroides* in both the AIS-T and CON-T groups (Fig. S4D and E). LEfSe analysis showed that *Parabacteroides*, *Oscillospira*, *Morganella*, and *Dialister* were significantly higher in the AIS-T group, whereas *Roseburia* and *Coprococcus* were significantly higher in the CON-T group (Fig. 6A). Random forest analysis showed that *Roseburia* was the most critical genus in the training cohort to discriminate AIS patients from healthy controls. Spearman correlation analysis showed that *Roseburia* abundance was significantly negatively correlated with fibrinogen (*R* = −0.365, *P* = 0.009) and D-dimer (*R* = −0.458, *P* = 0.001) levels in the AIS-T group (Fig. 6B).

To construct the best combination of gut microbial biomarkers to discriminate AIS patients from healthy controls, we used cross-validation analysis. When we used the 15 most significantly different bacteria between AIS patients and healthy controls for modeling, the cross-validation error was minimized (Fig. S4F), and the model exhibited high predictive stability and reproducibility (Fig. S4G). Therefore, we constructed ROC curves using these 15 bacteria as combinations of gut microbial biomarkers. These gut microbial biomarkers showed good performance in discriminating AIS patients from healthy controls, with AUC values of 0.939 and 0.901 in the training and validation cohorts, respectively (Fig. 6C). We further investigated the performance of coagulation indices (D-dimer and fibrinogen) and gut microbial biomarkers in predicting poor functional outcomes in AIS patients in the training and validation cohorts, respectively. Gut microbial biomarkers showed a good performance in predicting poor functional outcomes in AIS patients (training cohort: AUC = 0.930 and validation cohort: AUC = 0.890), which was better than that of coagulation indices (training cohort: AUC = 0.691 and validation cohort: AUC = 0.751). In both the training and validation cohorts, the combination of coagulation indices with gut microbial biomarkers showed the highest AUC in predicting poor outcomes in AIS patients (training cohort: AUC = 0.943 and validation cohort: AUC = 0.944; Fig. 6D). Our results suggest that coagulation indices improved the predictive performance of gut microbial biomarkers for poor functional outcomes in AIS patients.

## DISCUSSION

The high prevalence and disability of acute ischemic stroke are a major medical burden worldwide (5, 6). AIS patients often exhibit hypercoagulable state along with gut microbiota dysbiosis. However, the relationship between hypercoagulable state and gut microbiota dysbiosis in AIS patients, as well as their potential predictive value for poor functional outcomes, has not been thoroughly investigated. Our study found that AIS patients showed hypercoagulable state with significantly elevated fibrinogen, which is an independent risk factor for poor functional outcomes in AIS patients at 90-day follow-up. For the first time, we found an association between gut microbiota dysbiosis and hypercoagulable state in AIS patients. AIS patients with hypercoagulable state showed significantly increased *Alistipes* and decreased *Prevotella*. More importantly, coagulation indices combined with gut microbial biomarkers showed better predictive performance for poor functional outcomes in AIS patients.

Host coagulation and fibrinolytic systems are in a dynamic equilibrium (25). In the presence of relatively excessive coagulation or impaired fibrinolytic function, disruption of the dynamic balance of the coagulation-fibrinolytic system increases the risk of thrombosis. AIS is a cerebrovascular disease associated with thrombosis (26), and fibrinogen-rich thrombi have been reported to be significantly associated with myocardial stroke (27), suggesting that significant activation of coagulation may accompany the pathophysiological process of AIS, including the onset, progression, and recovery of this disease. Our results showed that the levels of fibrinogen and D-dimer were significantly elevated in patients with AIS compared with healthy controls, which is consistent with previous studies (7, 8). Fibrinogen is a glycoprotein secreted by the liver that plays an important role in the process of thrombosis and procoagulation (28). D-dimer is a product of fibrin degradation and a biomarker of coagulation that promotes the thrombus formation process (29, 30). The significant increase in fibrinogen and D-dimer levels after stroke indicates a significant activation of coagulation and a general hypercoagulable state in AIS patients. Similarly, Hou et al. found that high baseline and 90-day fibrinogen levels were associated with poor long-term outcomes in ischemic stroke and transient ischemic attack patients (31). Therefore, AIS patients were generally hypercoagulable with significantly elevated fibrinogen levels, an independent risk factor for poor functional outcomes.

Our study found that the α-diversity indices Chao1 and ACE were significantly higher in AIS patients compared to healthy controls, indicating a significant increase in gut microbiome richness in AIS patients. Consistent with previous studies, Yin et al. reported that the Chao1 index and observed species were significantly higher in AIS and transient ischemic attack patients compared to healthy controls (32). A clinical study by Xiang et al. also showed that both the ACE and Chao1 indices were significantly higher in AIS patients compared to healthy controls (33). AIS patients showed significant changes in the composition of the gut microbiota (34–37). In our study, the abundance of *Parabacteroides* was significantly higher, while the abundance of *Prevotella*, *Roseburia*, and *Faecalibacterium* was significantly lower in AIS patients, which is consistent with the previous studies of Yin and Li et al. (32, 38). Several clinical studies also reported a decrease in *Roseburia* abundance in stroke patients (38–40). A clinical study by Li et al. included 79 patients with AIS and 98 controls and showed a significant decrease in *Roseburia* abundance in AIS patients (38).

Our study observed an association between gut microbiota dysbiosis and a hypercoagulable state in AIS patients. *Parabacteroides* enriched in patients with AIS was found to be significantly and positively correlated with APTT, and *Alistipes* was significantly and positively correlated with fibrinogen. Previous studies have reported that *Parabacteroides* was involved in promoting the progression of risk factors for AIS. For example, *Parabacteroides distasonis* was enriched in patients with gestational diabetes mellitus and positively correlated with blood glucose levels (41). *Parabacteroides distasonis* abundance was significantly increased in patients with coronary artery disease and was significantly positively correlated with total cholesterol and low-density lipoprotein levels (42). In addition, *Alistipes* was significantly enriched in hypertensive patients, with its abundance positively correlated with systolic blood pressure levels, potentially contributing to intestinal barrier dysfunction and inflammation (43). We also found that *Roseburia* and *Prevotella*, which were significantly reduced in AIS patients, had negative associations with coagulation indices. Specifically, *Prevotella* showed a strong negative correlation with APTT, while *Roseburia* was negatively correlated with fibrinogen and D-dimer. In healthy individuals, *Prevotella copri* is significantly negatively associated with fasting very-low-density lipoprotein levels and postprandial blood glucose levels (44). Higher levels of *Roseburia* are associated with a lower incidence of type 2 diabetes (45). Colonization of atherosclerosis-prone ApoE^–/–^ sterile mice with *Roseburia* reduces inflammatory markers and ameliorates atherosclerosis (46). In addition, Gu et al. found a significant negative correlation between *Roseburia* abundance and NIHSS scores as well as prognostic mRS scores in AIS patients (47). Future clinical and basic studies are warranted to further explore the associations and mechanisms of *Parabacteroides*, *Alistipes*, *Prevotella*, and *Roseburia* with coagulation function.

AIS patients reflected significantly increased *Alistipes* abundance with decreased *Prevotella* abundance compared to healthy controls. Furthermore, the AIS-HC group showed an even higher *Alistipes* abundance with a significant decrease in *Prevotella* abundance compared to the AIS-NC group. In the PICRUSt2 analysis and correlation analysis, the 1,4-dihydroxy-6-naphthoate biosynthesis pathway was upregulated in AIS-HC patients and positively correlated with *Blautia* and *Alistipes*, which were significantly enriched in the AIS-HC group. 1,4-Dihydroxy-6-naphthoate is a key component of the gut microbiota biological pathway for the synthesis of menaquinone (48). Menaquinone, also known as vitamin K2, promotes the hepatic synthesis of coagulation factor II and regulates the synthesis of coagulation factors VII, IX, and X *in vivo* (49). Pyridoxal 5'-phosphate biosynthesis and the pentose phosphate pathway were significantly upregulated in AIS-HC patients, and both were negatively correlated with *Prevotella* and *Lachnospira*, which were significantly reduced in the AIS-HC group. Pyridoxal 5'-phosphate, as the active form of vitamin B6, is involved in homocysteine (Hcy) metabolism (50). Vitamin B6 deficiency can lead to elevated Hcy levels, increasing the risk of cardiovascular disease and atherosclerosis (51). The pentose phosphate pathway primarily synthesizes nicotinamide adenine dinucleotide phosphate (NADPH), leading to an increase in reactive oxygen species, which further promotes platelet activation, aggregation, granule secretion, and procoagulant activity (52). The L-1,2-propanediol degradation pathway, which was significantly downregulated in the AIS-HC group, was negatively correlated with *Alistipes*. The L-glutamate to propanoate pathway, which was significantly downregulated in the AIS-HC group, was positively correlated with *Prevotella* and *Megamonas*. The end product of the propanediol degradation pathway is propanoate (53). *Prevotella* breaks down arabinoxylan and oligofructose, leading to the production of high levels of propanoate (54, 55). Propanoate, as an important component of short-chain fatty acids, plays an important role in improving the intestinal barrier and alleviating intestinal inflammation (56). Propanoate also activates the G protein-coupled receptor GPR41 in the kidney and vascular smooth muscle to lower blood pressure (57). Therefore, in AIS patients with hypercoagulable state, increased *Alistipes* and decreased *Prevotella* are observed along with alterations in gut microbiota-related pathways, warranting further research on their potential impact on coagulation status.

Gut microbiota can be used as biomarkers to predict clinical outcomes in AIS. A clinical study by Xu et al. included 98 neurocritical care patients and found their significant gut microbiota dysbiosis compared to healthy controls. During hospitalization in the neurointensive care unit, stroke patients showed exacerbated gut microbiota dysbiosis, which was correlated with their 180-day mortality (58). Xia et al. calculated a stroke dysbiosis index (SDI) based on 18 bacterial genera that differed between stroke patients and healthy controls. The SDI was significantly and positively correlated with stroke patients mRS scores at discharge, and the SDI was an independent risk factor for poor functional outcomes in AIS patients at 90-day follow-up (11). According to a clinical study by Shi et al., a predictive model based on characteristic microbiota (*Romboutsia* and *Fusicatenibacter*) had AUC values of 0.7193 and 0.6839 for predicting poor functional outcome at 3 months in AIS patients (59). By combining these two genera and three clinical parameters (NIHSS score, Hcy, and Hamilton Depression Scale), the AUC reached the highest value of 0.8636, which is comparatively lower than the AUC values of predictive models in our study (training cohort: AUC = 0.943 and validation cohort: AUC = 0.944). Our study found that gut microbial biomarkers accurately predicted poor functional outcomes in AIS patients with better performance compared to coagulation indices. Coagulation indices combined with gut microbial biomarkers showed the highest predictive performance for poor functional outcomes in AIS patients.

In conclusion, our study confirms that AIS patients with hypercoagulable state had significantly elevated fibrinogen levels that independently predicted poor functional outcomes, supporting the clinical utility of fibrinogen in the prognostic management of AIS patients. In addition, AIS patients with hypercoagulable state showed a significant increase in *Alistipes* and a decrease in *Prevotella*, which were significantly associated with post-stroke hypercoagulable state, providing a potential novel target for subsequent improvement of the hypercoagulable state. Importantly, we develop a novel combination of coagulation indices with gut microbial biomarkers that show promising predictive performance for poor functional outcomes in AIS patients at 90 days post-stroke. Future multicenter clinical trials are warranted to further validate the predictive value of gut microbial biomarkers combined with coagulation indices in AIS patients. The mechanisms by which gut microbiota dysbiosis lead to hypercoagulable state need to be investigated in future studies.

## Data Availability

The raw data for the 16S rRNA gene sequences in this study are available from the Sequence Read Archive (https://www.ncbi.nlm.nih.gov/bioproject) at accession number PRJNA1180644. The Strengthening the Organizing and Reporting of Microbiome Studies (STORMS) checklist is available at https://doi.org/10.5281/zenodo.13364212.

## EDITOR'S NOTE

Ed. Note: A conflict of interest was identified after acceptance of this paper. As a result, the manuscript was transferred to a senior editor and the editor in chief, who arranged for a third review and handled the final decision.

## SUPPLEMENTAL MATERIAL

Supplemental files msystems.01492-24-s0001.pdf Figures S1-S4; Tables S1-S3.

ASM does not own the copyrights to Supplemental Material that may be linked to, or accessed through, an article. The authors have granted ASM a non-exclusive, world-wide license to publish the Supplemental Material files. Please contact the corresponding author directly for reuse.
