# Supplementary material for: Hypercoagulable state and gut microbiota dysbiosis as predictors of poor functional outcomes in acute ischemic stroke patients
Source: mSystems. 2025 Apr 9;10(5):e01492-24. doi: 10.1128/msystems.01492-24 (PMC12090755; doi:10.1128/msystems.01492-24)
Supplement: Supplemental files — Figures S1-S4; Tables S1-S3. [file msystems.01492-24-s0001.pdf]

Supplementary materials for

Hypercoagulable State and Gut Microbiota Dysbiosis as Predictors of Poor Functional Outcomes in Acute Ischemic Stroke Patients

Authors: Jie Li[1]*, Shengnan Chen[1]*, Siqi Yang[1]*, Wen Zhang[1], Xiaoqi Huang[1], Lang Zhou[1], Yanchao Liu[2], Mengxi Li[1], Yonghui Guo[1], Jia Yin[3†], Kaiyu Xu[1†]

Affiliations:

[1]Microbiome Medicine Center, Department of Laboratory Medicine, Zhujiang Hospital, Southern Medical University, Guangzhou, Guangdong, 510282, China.

[2]Department of Neurosurgery Center, Zhujiang Hospital, Southern Medical University, Guangzhou, Guangdong, 510282, China.

[3]Department of Neurology, Nanfang Hospital, Southern Medical University, Guangzhou, Guangdong, 510515, China.

* These authors contributed equally to this work.

† Correspondence to:

Kaiyu Xu, Microbiome Medicine Center, Department of Laboratory Medicine, Zhujiang Hospital, Southern Medical University, Guangzhou, Guangdong, 510282, China; Email: xukaiyu09@smu.edu.cn

Jia Yin, Department of Neurology, Nanfang Hospital, Southern Medical University, Guangzhou, Guangdong, 510515, China; Email: yinj@smu.edu.cn

This file includes:

Supplementary figures S1-S4

Supplementary tables S1-S3

Supplementary figures S1-S4

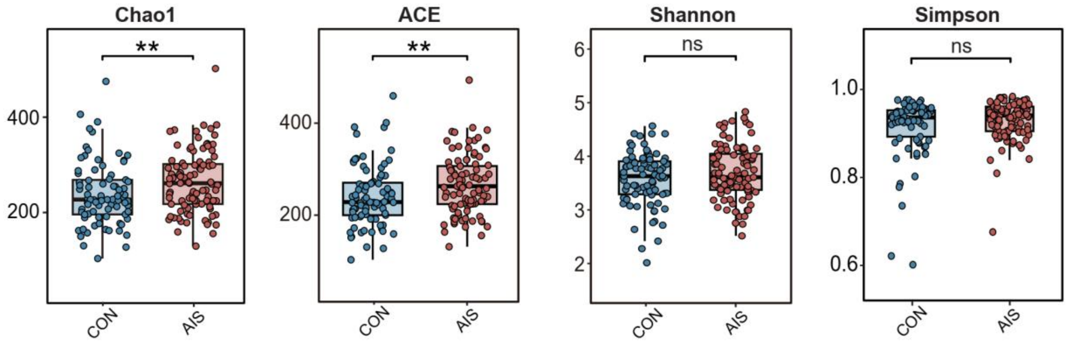

Fig. S1 Alpha diversity of gut microbiota (Chao1, ACE, Shannon and Simpson indices) between AIS patients and healthy controls. *p<0.05, **p<0.01.

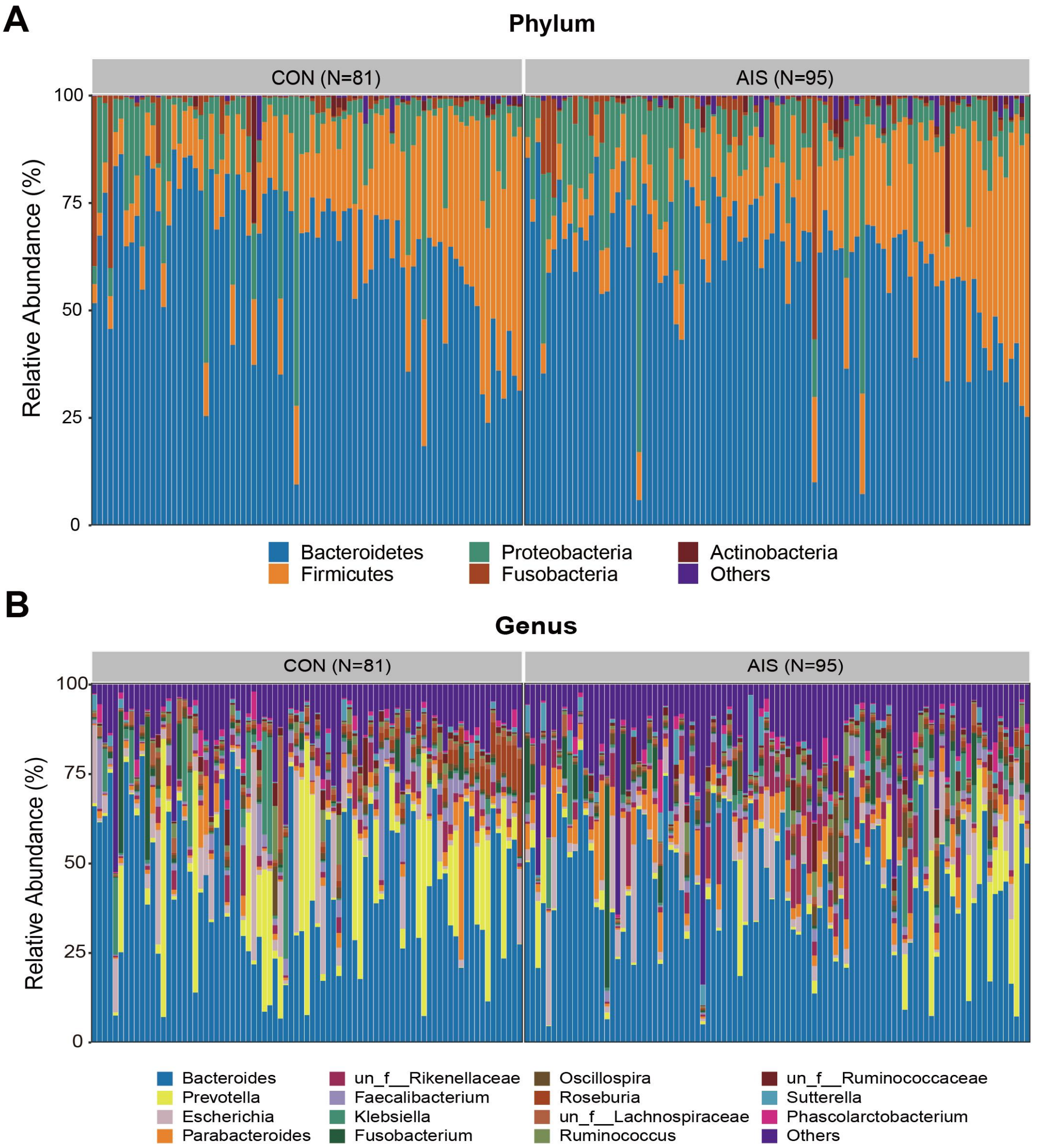

Fig. S2 The relative abundance of gut microbiota in individual AIS patients and healthy controls at the phylum level (A) and genus level (B).

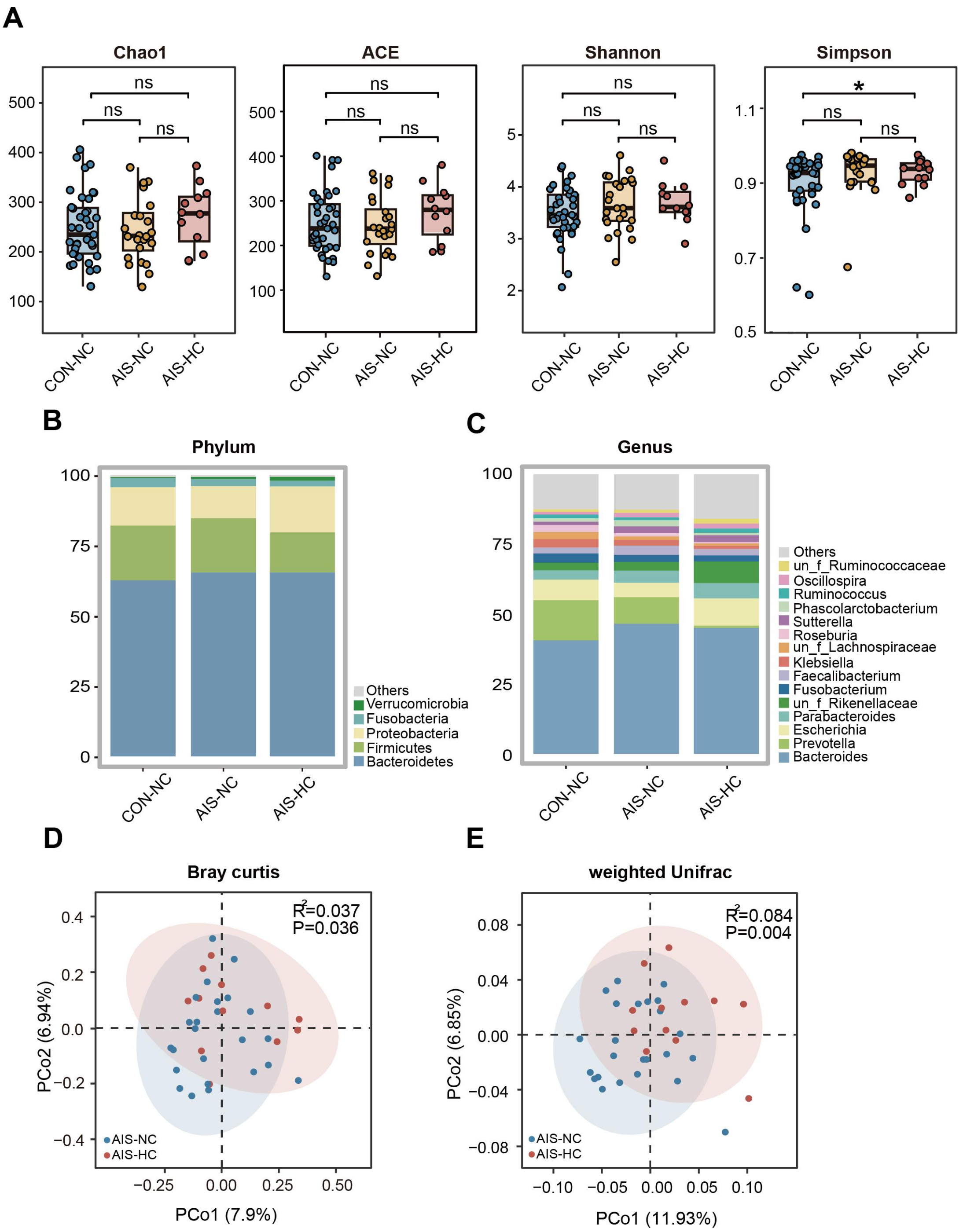

Fig. S3 Composition of gut microbiota among CON-NC, AIS-NC and AIS-HC groups. (A) Alpha diversity (Chao1, ACE, Shannon and Simpson indices) of gut microbiota among CON-NC, AIS-NC and AIS-HC groups. (B-C) Gut microbial composition at phylum and genus level in CON-NC, AIS-NC and AIS-HC groups. (D-E) PCoA plots based on Bray-Curtis and weighted Unifrac showing significant gut microbiota alteration in the

AIS-NC group compared to the AIS-HC group. CON-NC group, healthy controls with normal coagulable function; AIS-NC group, AIS patients with normal coagulable function; AIS-HC group, AIS patients with hypercoagulable state. *$p<0.05$.

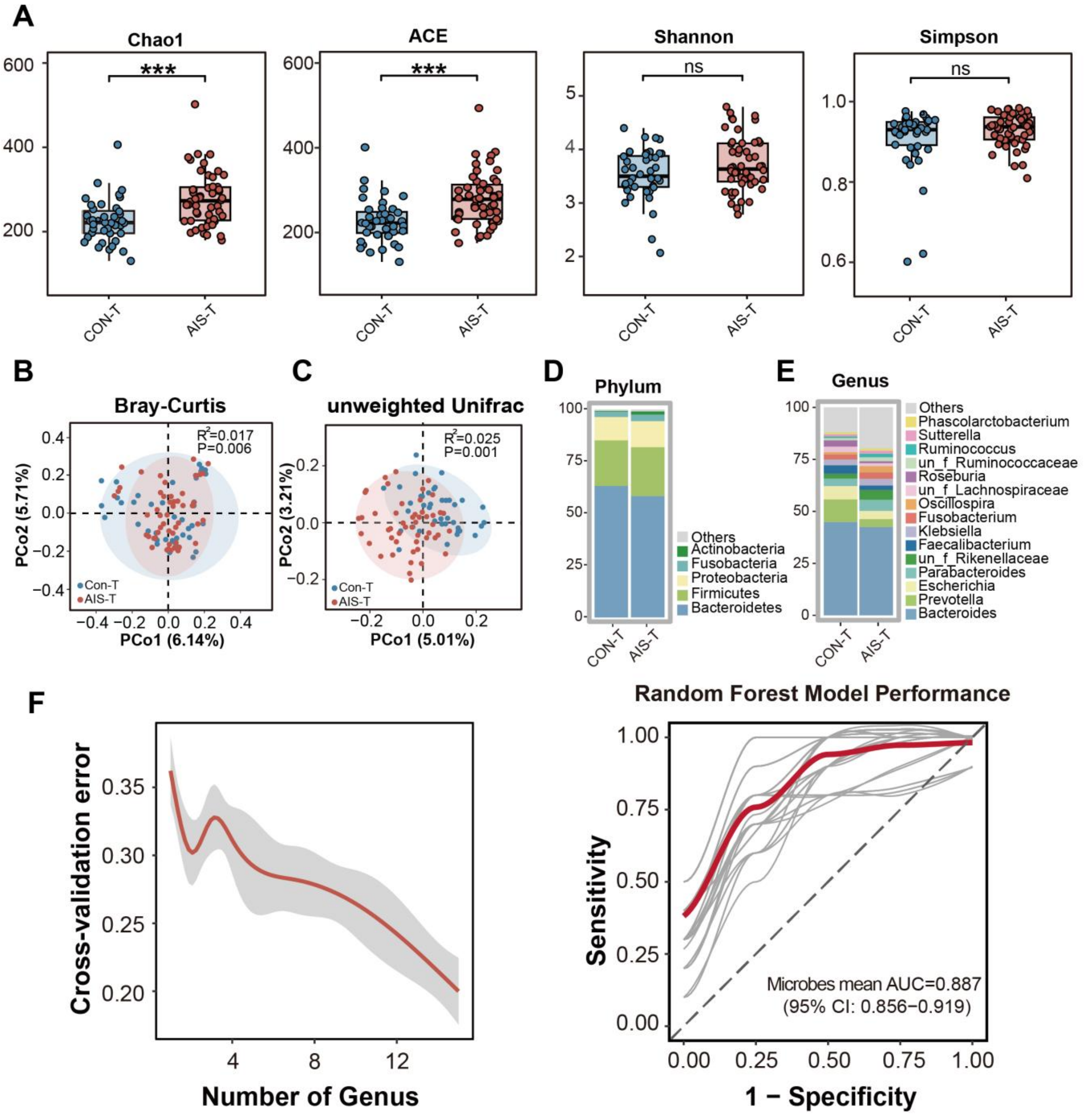

Fig. S4 AIS-T group showed significant gut microbiota dysbiosis compared with CON-T group. (A) Alpha diversity (Chao1, ACE, Shannon and Simpson indices) of the gut microbiota between AIS patients and healthy controls in the training cohort. (B-C) PCoA plots based on Bray-Curtis and unweighted Unifrac showing significant gut microbiota alteration in the AIS-T group compared to the CON-T group. (D-E) Gut microbial composition at phylum and genus level in AIS-T group and CON-T group. (F) Cross-validation curves for screening gut microbial biomarkers in the training cohort using

random forest analysis. (G) Cross-validation of the ROC curve with 95%CI demonstrated the stability and reproducibility of the predictive model. AIS-T, AIS patients in training cohort; CON-T, healthy controls in training cohort; ROC, receiver operating characteristic; CI, confidence interval; ***p<0.001.

Supplementary tables S1-S3

Table S1 Characteristics of AIS patients with good and poor functional outcome.

Characteristics	AIS patients with good outcome	AIS patients with poor outcome	P value
Number	68	27	/
Age, years	65.50(14.00)	71.00(16.00)	0.015
Male, n (%)	43(63.20)	14(51.90)	0.307
Hypertension, n (%)	46(67.60)	21(77.80)	0.329
Diabetes, n (%)	17(25.00)	8(29.60)	0.644
Coronary heart disease, n (%)	5(7.40)	2(7.40)	1.000
Smoking, n (%)	8(11.80)	3(11.10)	1.000
WBC, 10^9/L	7.46(3.16)	8.26(5.15)	0.134
RBC, 10^{12}/L	4.66(0.79)	4.52(1.04)	0.473
HGB, g/L	137.50(23.00)	130.00(30.00)	0.265
PLT, 10^9/L	229.00(71.00)	228.00(95.00)	0.987
ALT, IU/L	15.00(11.00)	14.00(11.00)	0.121
AST, IU/L	18.50(8.00)	18.00(7.00)	0.418
Cr, μmol/L	77.00(23.81)	71.47(30.00)	0.236
Glu, mmol/L	6.36(2.26)	7.14(3.19)	0.967
PT, s	11.30(1.00)	11.70(1.80)	0.010
APTT, s	25.40(2.98)	25.50(2.70)	0.483
TT, s	16.10(1.08)	15.50(1.50)	0.033
DDI, mg/L	0.35(0.34)	0.88(1.18)	0.003

Fg, g/L	2.92(1.13)	3.68(1.57)	0.021

Continuous variables are expressed as medians (IQR), and categorical variables are expressed as frequencies (percentages). WBC, white blood cells; RBC, red blood cells; HGB, hemoglobin; PLT, platelets; ALT, alanine aminotransferase; AST, aspartate transaminase; Cr, creatinine; Glu, glucose; PT, prothrombin time; APTT, activated partial thromboplastin time; TT, thrombin time; DDI, D-dimer; Fg, fibrinogen.

Table S2 Univariate and Multivariate Regression Analysis for Predicting Functional Outcomes in AIS Patients.

Variable	Univariate analysis		Multivariate analysis	
	OR (95% CI)	*P* value	OR (95% CI)	*P* value
Clinical indices				
Age	1.06 (1.01, 1.11)	0.010	1.10 (1.02, 1.19)	0.009
Sex	1.60 (0.65, 3.94)	0.309	-	-
Hypertension	1.67 (0.59, 4.74)	0.332	-	-
Diabetes	1.26 (0.47, 3.41)	0.644	-	-
CHD	1.01 (0.18, 5.54）	0.993	-	-
Smoking	0.94 (0.23, 3.84)	0.928	-	-
WBC	1.14 (0.99, 1.31)	0.077	1.03 (0.86, 1.24)	0.741
PLT	1.00 (0.99, 1.01)	0.961	-	-
Glu	0.93 (0.82, 1.07)	0.323	-	-
Cr	0.99 (0.97, 1.01)	0.400	-	-
PT	2.06 (1.21, 3.50)	0.008	1.04 (0.49, 2.21)	0.924
APTT	1.05 (0.88, 1.25)	0.615	-	-
TT	0.74 (0.48, 1.13)	0.160	-	-
Fg	1.90 (1.22, 2.94)	0.004	2.16 (1.02, 4.59)	0.044
DDI	1.96 (1.19, 3.23)	0.008	1.07 (0.56, 2.06)	0.834
NIHSS	1.23 (1.11, 1.38)	0.001	1.31 (1.14, 1.49)	0.001

CHD, coronary heart disease; WBC, white blood cells; PLT, platelets; Glu, glucose; Cr, creatinine; PT, prothrombin time; APTT, activated partial thromboplastin time; TT, thrombin time; DDI, D-dimer; Fg, fibrinogen; NIHSS, National Institutes of Health Stroke Scale.

Table S3 Characteristics of study participants in the CON-T, AIS-T, CON-V and AIS-V groups [a].

Characteristics	Training cohort		Validation cohort		P value
	CON-T	AIS-T	CON-V	AIS-V	
Number	40	50	41	45	/
Age, years	67.50(8.75)	67.50(17.75)	62.00(13.00)	66.00(12.50)	0.140
Male, n (%)	21(52.50)	27(54.00)	23(56.10)	30(66.70)	0.525
Hypertension, n (%)	16(40.00)	37(74.00)[b]	11(26.80)	30(66.70)[c]	<0.001
Diabetes, n (%)	3(7.50)	13(26.00)[b]	2(4.90)	12(26.70)[c]	0.005
Coronary heart disease, n (%)	1(2.50)	4(8.00)	0	3(6.70)	0.252
Smoking, n (%)	2(5.00)	7(14.00)	3(7.30)	4(8.90)	0.543
WBC, 10^9/L	5.80(2.86)	7.69(3.37)[b]	6.63(2.06)	7.85(3.89)[c]	0.007
RBC, 10^{12}/L	4.51(0.71)	4.53(0.73)	4.49(0.52)	4.67(0.90)	0.659
HGB, g/L	133.50(19.50)	134.50(23.50)	134.00(20.00)	138.00(24.00)	0.953

PLT, 10^9/L	227.00(65.00)	230.50(66.50)	243.00(95.50)	220.00(88.00)	0.570
ALT, IU/L	16.00(7.75)	14.00(14.25)	16.00(10.000	15.00(6.50)	0.610
AST, IU/L	18.00(7.00)	19.00(7.50)	18.00(8.00)	18.00(5.00)	0.238
Cr, μ mol/L	66.50(30.75)	77.00(24.75)	74.00(22.00)	75.00(26.19)	0.461
Glu, mmol/L	5.25(0.68)	6.72(3.25)[b]	4.89(0.91)	6.46(3.30)[c]	<0.001
PT, s	11.30(0.97)	11.40(1.40)	11.30(0.80)	11.50(0.90)	0.831
APTT, s	25.90(2.55)	25.65(2.85)	25.90(2.50)	25.20(3.15)	0.671
TT, s	16.00(0.78)	16.20(1.35)	16.20(1.15)	15.90(1.10)	0.399
DDI, mg/L	0.36(0.24)	0.51(0.90)[b]	0.29(0.29)	0.38(0.43)	0.064
Fg, g/L	2.81(0.94)	3.03(1.05)[b]	2.83(0.81)	2.83(1.59)	0.020

[a] Continuous variables are expressed as medians (IQR), and categorical variables are expressed as frequencies (percentages). WBC, white blood cells; RBC, red blood cells; HGB, hemoglobin; PLT, platelets; ALT, alanine aminotransferase; AST, aspartate transaminase; Cr, creatinine; Glu, glucose; PT, prothrombin time; APTT, activated partial thromboplastin time; TT, thrombin time; DDI, D-dimer; Fg, fibrinogen. AIS-T, AIS patients in training cohort; CON-T, healthy controls in training cohort; AIS-V, AIS patients in validation cohort; CON-V, healthy controls in validation cohort.

[b] $P<0.05$ when AIS-T group compared with CON-T group.

[c] $P<0.05$ when AIS-V group compared with CON-V group.
